# Fluorescent Phase-Changing Perfluorocarbon Nanodroplets as Activatable Near-Infrared Probes

**DOI:** 10.3390/ijms23137312

**Published:** 2022-06-30

**Authors:** Catalina-Paula Spatarelu, Austin Van Namen, Sidhartha Jandhyala, Geoffrey P. Luke

**Affiliations:** 1 Dartmouth College, 15 Thayer Drive, Hanover, NH 03755, USA; catalina-paula.spatarelu.th@dartmouth.edu (C.-P.S.); acvnth@gmail.com (A.V.N.); sidhartha.jandhyala.th@dartmouth.edu (S.J.); 2 Norris Cotton Cancer Center, 1 Medical Center Drive, Lebanon, NH 03766, USA

**Keywords:** multimodal imaging, ultrasound-enhanced fluorescence, NIR probes

## Abstract

The sensitivity of fluorescence imaging is limited by the high optical scattering of tissue. One approach to improve sensitivity to small signals is to use a contrast agent with a signal that can be externally modulated. In this work, we present a new phase-changing perfluorocarbon nanodroplet contrast agent loaded with DiR dye. The nanodroplets undergo a liquid-to-gas phase transition when exposed to an externally applied laser pulse. This results in the unquenching of the encapsulated dye, thus increasing the fluorescent signal, a phenomenon that can be characterized by an ON/OFF ratio between the fluorescence of activated and nonactivated nanodroplets, respectively. We investigate and optimize the quenching/unquenching of DiR upon nanodroplets’ vaporization in suspension, tissue-mimicking phantoms and a subcutaneous injection mouse model. We also demonstrate that the vaporized nanodroplets produce ultrasound contrast, enabling multimodal imaging. This work shows that these nanodroplets could be applied to imaging applications where high sensitivity is required.

## 1. Introduction

Fluorescence imaging is a nonionizing modality that is ubiquitous in biomedical applications, with applications ranging from diagnosis to interventional guidance [1]. It can provide information on the physiological state of tissues, as well as the molecular environment. As a result, fluorescent imaging is an important tool in cancer diagnosis and treatment. Fluorescence imaging can be employed intraoperatively, in the form of fluorescent-guided surgery procedures that aim to improve tumor margins [2], as well as perioperatively for characterizing biopsy samples for biomarker expression or sentinel lymph node mapping [3,4].

Due to the strong scattering and absorption of visible light in tissues, however, fluorescence imaging is generally restricted to superficial applications or ex vivo measurements [5]. To address this, fluorophores in the near-infrared (NIR) range (700–900 nm), and, more recently, in the NIR-II window (1000–1700 nm), have been proposed as alternatives [1,6,7,8,9]. In these wavelength windows, photon absorption, scattering and autofluorescence are decreased, translating into higher penetration depths compared to visible light [7]. Unfortunately, even the NIR-II probes proposed to date are still restricted to sub-centimetric applications in vivo [6], and are unable to achieve a spatial resolution that allows for microscopic information in deep tissues [10].

In an effort to improve sensitivity to small concentrations of fluorescent contrast agents, externally activatable probes have been developed [6,11]. The mechanism behind switching the fluorescence on and off relies on the propensity of fluorophores to quench under certain conditions and unquench in response to a specific stimulus [6]. Several options for externally triggered systems have been investigated, including temperature-sensitive nanoparticles, light-activatable probes or ultrasound-sensitive nanoparticles [11,12,13,14,15]. Ultrasound is an appealing energy source for the external activation of fluorophores because of its good penetration and minimal scattering in soft tissue. A handful of ultrasound-sensitive agents have been demonstrated to improve the signal-to-noise ratio of fluorescence images [10,16]. Because of the ability to focus ultrasound into a relatively small volume in tissue, the techniques have an added benefit of improved imaging resolution [17].

Ultrasound-sensitive fluorescent agents can be synthesized in either microbubble [16,18] or nanoparticle forms [10,13,19,20]. Microbubbles expand and contract in response to incident acoustic waves. This can lead to increased and decreased spacing between encapsulated fluorescent molecules. Thus, rapid quenching and unquenching is achieved, yielding a high-frequency modulated fluorescent signal. However, microbubbles are limited due to their size, and normally cannot extravasate into tissue, providing information from the vasculature only [21]. Their micrometer-scale size also makes their circulation times short, usually on the order of minutes, which limits the window for imaging.

In contrast to microbubbles, nano-encapsulated fluorophores benefit from extended circulation times and enhanced tumor accumulation and retention [22]. These features make them attractive to be used as ultrasound-switchable probes. Recently, thermo-sensitive polymeric particles have been developed to respond to temperature increase due to high-intensity focused ultrasound [17,23]. This technique managed to achieve large ON/OFF ratios; however, the temperature thresholds needed are on the order of 10 °C, which could lead to localized tissue damage. Phase-changing nanodroplets, containing a perfluorocarbon core that can be vaporized with an externally applied acoustic or laser pulse, have been used as microbubble precursors in several applications, including ultrasound and photoacoustic imaging [24,25], image-guided drug delivery [26,27] or opening the brain–blood barrier [21,28]. By relying on a liquid-to-gas phase change, the drawbacks of temperature-responsive agents could be avoided. Our work herein investigates the feasibility of using such a platform as a switchable NIR fluorescent probe.

This study consists of the design and characterization of a 1,1′-dioctadecyl-3,3,3′,3′-tetramethylindotricarbocyanine iodide (DiR)-loaded phase-changing nanodroplet platform that is optically activatable. The transition from liquid-core nanodroplets (“OFF”) to gaseous-core microbubbles (“ON”) was leveraged for unquenching the loaded DiR for an increase in the fluorescence intensity and signal/background ratio.

## 2. Results and Discussion

**Figure 1:**
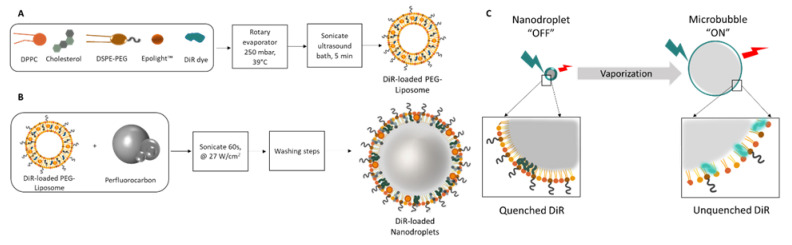
(**A**) Thin-layer evaporation method to obtain DIR-loaded liposome; (**B**) mixing preformed liposomes with perfluoropentane to obtain DiR-loaded PFP nanodroplets; (**C**) schematic of the switchable fluorescent nanodroplets going from the nanodroplet state to the microbubble state due to vaporization of the core. The transition causes quenched DiR encapsulated into the nanodroplets to partially unquench, resulting in an increase in fluorescence intensity.

DiR-loaded nanodroplets were obtained by a thin-layer evaporation technique (Figure 1A,B), using a mixture of DSPE-PEG_2000_, DPPC and cholesterol in a molar ratio of 15:35:50. Epolight 3072™ was used to impart the NIR light sensitivity of droplets. Due to the presence of Epolight™, the nanodroplets can be externally vaporized using pulsed NIR laser light (Figure 1C), generating microbubbles with a five-fold increase in size. We hypothesized that the DiR could be loaded in nanodroplets in a quenched state due to molecule proximity; after activation, the dye molecule separation increases because of the increase in the size of the lipid membrane, becoming partially unquenched. The nanodroplets therefore correspond to the “OFF” state, while microbubbles are in an “ON” state, with the ratio between the fluorescence of the two states an important measurement to characterize.

**Figure 2:**
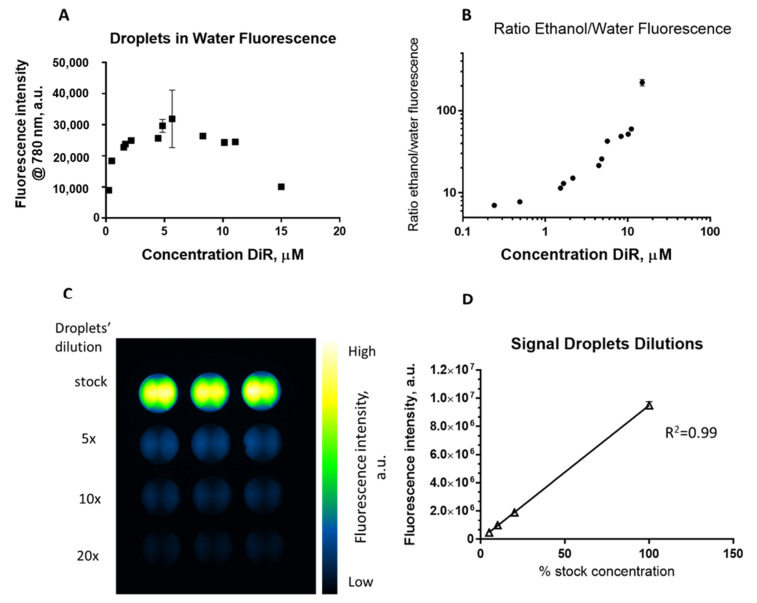
(**A**) Fluorescence of DiR-loaded droplets dispersed and measured in water; (**B**) ratio of fluorescence of unquenched, DiR dye in destroyed droplets and quenched, nanodroplet-encapsulated DiR from water-dispersed nanodroplets; (**C**) Pearl Impulse fluorescent image of DiR-loaded nanodroplets—1.90 μM stock—in serial dilutions in water; (**D**) fluorescence intensity of DiR-loaded nanodroplet stock dilutions.

In order to test this hypothesis, a range of DiR concentrations were encapsulated in the nanodroplets. Nanodroplets with a size of ~400 nm and good stability at 37 °C for at least 4 h were obtained (Figure S1). The fluorescence intensity was measured with a spectrofluorometer (Fluoromax, Horiba, Kyoto, Japan). To measure the extent of the quenching, the nanodroplets were measured as dispersed in water—to determine the fluorescence of the quenched dye—and measured after they were destroyed in ethanol—to determine the fluorescence of the unquenched dye. One aspect to note is that the quantum yields of DiR in the lipid environment and ethanol are different, so even for droplets containing DiR in an unquenched state, the fluorescence intensity of the encapsulated dye will not be equal to the fluorescence intensity of unencapsulated dye (Figure S2). While the intensity of fluorescence increases with the amount of dye encapsulated in the destroyed nanodroplet samples, the water samples show the quenching effect at µM concentrations (Figure 2A). The signal of nanodroplet-encapsulated DiR dispersed in water does not increase linearly anymore, compared to the signal in ethanol of the unquenched dye. This difference can be noticed when computing the ratio between the fluorescence of droplet samples in ethanol to that of droplets dispersed in water (Figure 2B). To study whether the quenching of nanodroplet-loaded DiR occurs through intraparticle or interparticle interactions, a single batch of nanodroplets with 1.9 µM encapsulated DiR was used to create a dilution series of undiluted droplets (“stock”), followed by dilutions to 5×, 10× and 20×. The fluorescence was measured using a fluorescence imaging instrument (Pearl Impulse, LI-COR), using an excitation of 785 nm and an emission filter of 820 nm. The diluted samples showed a linear increase in signal up to the stock concentration of droplets (Figure 2C,D). This indicates that the quenching effect happens intraparticle, rather than arising from particle–particle interactions.

**Figure 3:**
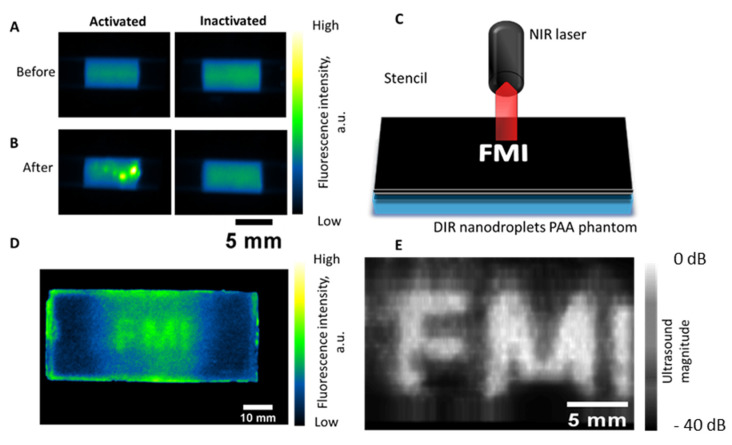
(**A**,**B**) The 20x diluted nanodroplets (5.87 μM) dispersed on either end of a plastic tube without contact between the parts before (**A**) and immediately after applying a laser pulse (**B**), where one end of the tube serves as inactivated control to observe any changes in fluorescence not due to the activation; (**C**) schematic showing the activation of a specific region of DiR-loaded nanodroplet polyacrylamide phantom while shielding the rest of the phantom; (**D**) florescence image of activated “FMI” phantom showing the enhanced fluorescence in the activated region; (**E**) 2D reconstruction of the ultrasound images of the activated “FMI” phantom.

In order to capture the fluorescence enhancement from the quenched nanodroplets’ activation, two aliquots of nanodroplets were drawn up into a 50 mm long, 4 mm outside diameter plastic tube with a small air gap between the two aliquots (Figure 3A,B). The concentration of the dye in the droplets was chosen to be in the middle of the studied range, at a value of 5.87 μM DiR dye encapsulated in the stock nanodroplet solution. Images were captured with the Pearl Impulse imaging system using an excitation of 785 nm and an emission filter of 820 nm. The tube was imaged before activation, and after activating one of the aliquots with a single laser pulse at 1064 nm with a fluence of 15 mJ/cm^2^, with the two instances being denoted “OFF” and “ON”, respectively.

To further confirm this phenomenon, the same nanodroplets were encapsulated in a polyacrylamide phantom. The phantom was activated using a 15 mJ/cm^2^ pulsed laser operating at 1064 nm (Phocus Mobile HE, Opotek) through a stencil spelling “FMI” (Figure 3C). After the activation of the nanodroplets with the laser, the fluorescence of the phantom was imaged (Figure 3D), indicating a fluorescence enhancement in the area of activated nanodroplets when compared to the background of inactivated nanodroplets in the same phantom. The letters appeared to have rather blurred edges, which we attribute to the diffraction of the beam from the optical bundle. To confirm that the nanodroplet vaporization resulted in microbubbles, which were highly echogenic and thus visible with ultrasound, a similar nanodroplet-laden phantom was obtained. The ultrasound image was obtained with a L22-8v Verasonics linear array ultrasound transducer connected to a Verasonics Vantage 256 imaging system. The lateral dimension spanned the 256 elements of the transducer (35 mm) and the imaging depth was 20 mm. The transducer was translated with a linear stage (TravelMax, Thor Labs) in 0.5 mm steps to achieve a three-dimensional image. The image was filtered with a 3 × 3 × 3 median filter and the signal was averaged along the depth direction to achieve a two-dimensional projection (Figure 3E). These results clearly indicate that the vaporization of nanodroplets with a pulsed laser increases the signal in both fluorescence and ultrasound images, enabling multimodal imaging. There was additional signal observed in both the fluorescence and ultrasound frames in non-activated regions after ultrasound exposure, which were most likely due to the acoustical activation of a small portion of the droplets.

**Figure 4:**
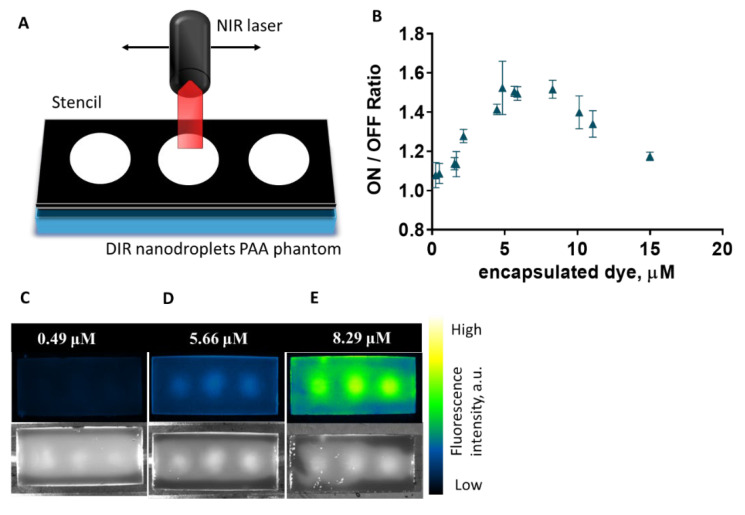
(**A**) Schematic of the activation of polyacrylamide phantoms containing DiR nanodroplets through a stencil with three circular openings; (**B**) ON/OFF ratio of fluorescence intensity of switchable DiR-loaded nanodroplets against total loaded DiR concentration; (**C**–**E**) PAA phantoms containing nanodroplets (3% volume of nanodroplets’ stock/v of phantom) with DIR loaded in different concentrations: 0.49 μM (**C**); 5.66 μM (**D**); 8.29 μM (**E**). The lower row shows the white light image of the same phantoms. All phantoms contained the same volumetric concentration of nanodroplets.

In order to quantify the enhanced fluorescence, a series of nanodroplet-laden phantoms was obtained using nanodroplets loaded with different dye concentrations, in the range of 0.24–15 μM. Each phantom was activated using a stencil with three circular spots (diameter = 15 mm) to allow for a good delimitation between activated and non-activated areas (Figure 4A). To gauge the degree of quenching of the loaded DiR, the ratio of fluorescence intensity in the activated area was normalized by the fluorescence intensity of the non-activated regions on the phantom. This “ON/OFF” ratio was computed using an average intensity across the three activated spots on each phantom, to account for any heterogeneity from activation. For each dye concentration, four phantoms with different nanodroplet volumetric concentrations were obtained, and the reported “ON/OFF” ratio is the average of the four different phantoms. Our hypothesis for the used range of DiR-loaded nanodroplets was that we could identify a lower limit, where the loaded DiR would be in an unquenched state and the activation would not cause a large difference in the “ON/OFF” ratio. Increasing the DiR encapsulated in nanodroplets was expected to result in an increase in the ratio, as the dye becomes more and more quenched as molecules are closer to one another in the nanodroplets. This was confirmed in our experiments (Figure 4B). Moreover, we observed a threshold above which the ratio of ON/OFF started to decrease again. We believe that this is due to the dye concentration being so high that even when the dye molecules grow farther apart in the formed microbubbles, they are so numerous that they are still in an aggregation-induced quenched state. Figure 4C–E show representative examples of activated phantoms containing DiR-encapsulating nanodroplets, where the DiR concentration in the stock batches is 0.49 μM, 5.66 μM and 8.29 μM, respectively. All the images have the same intensity map to allow for direct comparison.

**Figure 5:**
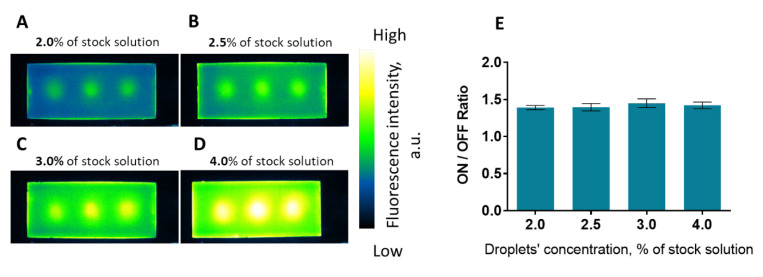
Polyacrylamide phantoms containing DIR-loaded nanodroplets (4.46 μM) with various volumetric concentrations of nanodroplets: 2.0% (**A**); 2.5% (**B**); 3.0% (**C**); 4.0% (**D**); all percentages are with respect to the stock concentration of nanodroplets; (**E**) graph showing the ratio between activated regions and background for phantoms shown in panels (**A**–**D**).

To determine whether interparticle quenching plays a role in the quenching/unquenching dynamics of DiR-loaded nanodroplets, several concentrations of the same droplets were used to obtain polyacrylamide phantoms as described before. Nanodroplets with a dye loading of 4.66 μM were chosen as they had shown a high ON/OFF ratio, indicating the dye being unquenched by nanodroplet vaporization. After activation, phantoms containing 2%, 2.5%, 3.0% and 4.0% nanodroplets’ stock suspension in the total phantom volume were measured using the same settings as in the previous experiments (Figure 5A–D). The results showed that the ratio was similar regardless of the volumetric concentration of nanodroplets in the phantoms (Figure 5E), with no statistically significant differences being recorded among the four phantoms. This indicates that the role of any interparticle phenomenon is negligible in this case, and only intraparticle dynamics come into play in the quenching and unquenching of nanodroplet-encapsulated DiR.

In order to confirm that the DiR nanodroplets can be used to increase the signal/background ratio of DIR upon droplet activation in vivo, a subcutaneous injection model was employed. Nude mice were injected into both flanks with a volume of 2 × 50 μL of a 5× dilution of DiR-loaded nanodroplets. Immediately after the injection, images were acquired and used to measure the fluorescence intensity in the two injection spots in the “OFF” state. Following the imaging, a NIR pulsed laser was applied onto only one of the spots, and an image recorded at the end of activation, denoted as the “ON” state. The non-activated spot was used as an internal control to account for changes independent of activation. The ON/OFF ratio was computed for each of the mice and plotted for both the non-activated spot and the activated one.

**Figure 6:**
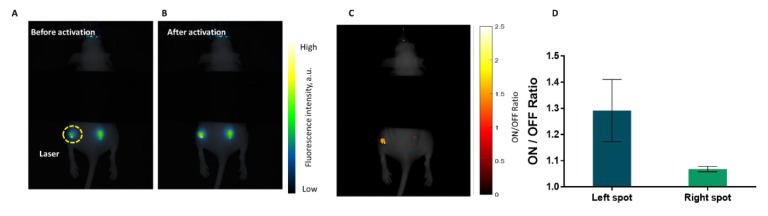
(**A**,**B**) Example image of a mouse in the subcutaneous study of the DiR-loaded nanodroplets before (**A**) and immediately after (**B**) pulsed laser activation. The yellow dashed line indicates the left spot on the mouse, which is activated, while the right one serves as an internal control. (**C**) Mouse from panel (**A**) with an overlay of ON/OFF ratios for pixels in both the left and the right spots; (**D**) average ON/OFF ratio for nanodroplets in both the non-activated spots, as well as activated ones. The “OFF” here indicates the signal intensity in nodes in the “before activation“ images and the “ON” is the signal intensity of the nodes in the “after activation”. Error bars indicate the standard deviation from 4 mice.

The results showed a significant increase in the signal intensity of activated spots compared to non-activated ones (Figure 6D), characterized by an ON/OFF ratio of 1.3 ± 0.12 and 1.07 ± 0.01, respectively (*p* = 0.025). This translates to a tenfold increase in the signal-to-background (SBR) in the activated spot for the image obtained by subtracting the two images, before and after activation. When looking at each individual mouse, results indicated that the change in signal in the non-activated spot was very similar between the samples, while the signal change in the case of the activated spot varied by a larger degree (Figure S3). This might be explained by the difference in the size of the injection for each of the mice. For situations where the bolus spread immediately after the injection, the activated area constituted a smaller fraction of the total area. This was in contrast with situations where the bolus remained more spatially concentrated, and the laser exposure covered most of the spot during activation (Figure 6A,B). For non-activated spots, this did not play a role, so the change was more uniform. These factors also explain the difference in the ratios observed in vivo compared to the phantom experiments, where the activated regions are clearly delimited. This can be observed to a certain degree when plotting the ON/OFF ratio for each pixel (Figure 6C). While the current setup does not allow for this, future studies will focus on the ability to gather fluorescence and ultrasound imaging simultaneously, to gain a greater resolution for the ON/OFF ratio.

## 3. Materials and Methods

### 3.1. Synthesis of DiR-Loaded Nanodroplets

Lipids were dissolved in chloroform and mixed together in a molar ratio of 1,2-dipalmitoyl-sn-glycero-3-phosphocholine (DPPC, NOF America):1, 2-Distearoyl-sn-glycero-3-phosphoethanolamine-Poly(ethylene glycol) (DSPE-PEG2000, NOF America, White Plains, NY, USA):Cholesterol (Alfa Aesar, Tewksbury, MA, USA) = 35:15:50. Epolight 3072 (Epolin, Newark, NJ, USA), with an activation maximum at 1064 nm added to impart the activatable capacity to nanodroplets. The fluorophore, 1,1′-dioctadecyl-3,3,3′,3′-tetramethylindotricarbocyanine iodide (DiR, Biotium, Fremont, CA, USA), was pre-dissolved in chloroform and added to the lipid and Epolight mixture. This mixture was evaporated under a 250 mbar vacuum at 39 °C until the formation of a thin film with a rotary evaporator (Heidolph North America, Wood Dale, IL, USA). The film was then rehydrated with distilled water and sonicated with a QSonica 700 tip probe sonicator, and perfluoropentane (Fluoromed, Round Rock, TX, USA) was added, followed by additional sonication. At the end of the synthesis, fluorescent nanodroplets were centrifuged and washed several times to remove unincorporated dyes or lipids, keeping them protected from the light to prevent photobleaching.

### 3.2. Fluorescence Measurement

Fluorescent nanodroplets were measured with a Fluoromax spectrofluorometer (HORIBA, Kyoto, Japan) with a 748 nm/780 nm setting for excitation/emission. To evaluate the amount of dye loaded, nanodroplets were centrifuged, the water supernatant discarded and replaced with ethanol. The sample was mixed until no turbidity could be observed, to ensure that all the nanodroplets and dye were dissolved in the ethanol. The samples were measured with a Fluoromax spectrofluorometer (HORIBA, Kyoto, Japan) with a 748 nm excitation wavelength and 780 nm emission. The concentration was computed by interpolation with a previously constructed calibration curve of fluorescence intensity against known DiR concentrations in ethanol.

### 3.3. In Vitro Studies of Laser-Triggered DiR Unquenching

Polyacrylamide (PAA) tissue-mimicking phantoms were obtained by a procedure described before. Briefly, a 10% polyacrylamide gel was prepared from a solution of acrylamide:bisacrylamide (29:1, Alfa Aesar, Tewksbury, MA, USA) adding up to 4.0% *v*/*v* nanodroplet suspension, and *N*,*N*,*N*′,*N*′-tetramethylethylendiamine (TEMED, Alfa Aesar, Tewksbury, MA, USA) and ammonium persulfate (APS, Sigma Aldrich, St. Louis, MO, USA) as an initiator system. To assess whether the concentration of droplets in phantoms plays a role in the unquenching effect, a range of concentrations were studied for each of the dye loadings. These concentrations ranged from 2% *v*/*v* with respect to the total phantom volume to 4% *v*/*v* with respect to the total phantom volume. This interval has been found to allow visualization of all samples with good contrast, including ones with lower dye loadings. Imaging was done using a Pearl Impulse imager (Li-Cor, Lincoln, NE, USA), using an excitation of 785 nm and measuring the emission at 820 nm. A plastic stencil with 3 circular holes of 15 mm diameter was used for the activation of spots on phantoms. Phantoms were imaged, and, for processing, the signal intensity inside the activation ROI was measured with ImageJ, as was the signal from the non-activated or “background” region. The ratio between the intensity of the signal in the activated spots and that of the background was denoted “signal/background” ratio and used to compare the samples in terms of unquenching.

### 3.4. Ultrasound Contrast

A polyacrylamide gel phantom was obtained as described above, with a 2% concentration of nanodroplets with respect to the total phantom volume. To gauge the ultrasound contrast capabilities of activated nanodroplets, a stencil was used to activate a region of the phantom, similar to the procedure described for the concentration study above. The main difference was that the stencil in this experiment spelled “FMI”, with the total height of a letter of 10 mm and width of 25 mm. The phantom was imaged with a L22-8v Verasonics linear array ultrasound transducer connected to a Verasonics Vantage 256 imaging system (Verasonics, Kirkland, WA, USA). B-mode ultrasound was collected by raster scanning the phantom beneath the transducer in the Y dimension. Each B-mode created an XZ plane, with the x dimension spanning the width of the 256 US transducer elements (35 mm) and the depth of the phantom (20 mm). In the Y-dimension, 27 images were taken at increments of 0.5 mm by translating the transducer with a linear stage (TravelMax, Thor Labs, Newton, NJ, USA). For the 2D image reconstruction, the image stack was first passed through a 5 × 5 × 5 3D median filter. The mean US signal was then averaged in the z-dimension, normalized to the maximum intensity and displayed as a single XY plane.

### 3.5. In Vivo Imaging of DiR-Loaded Nanodroplets

Female NU/NU nude mice (Charles River Laboratories, Wilmington, MA, USA) were used as an in vivo model. Images were acquired with the Li-Cor Pearl Impulse imaging system using the same settings as in the case of phantoms. DiR-loaded nanodroplets were injected subcutaneously in both hind sides of each mouse. Mice were sedated with 2.5% vol isofluorane (Fluoriso, VetOne, Boise, ID, USA) in O_2_ with a flow rate of 1.5 L/min for the duration of the procedure. Images were acquired prior to injection, immediately after injection and after pulsed laser activation of the droplets. One of the injection sites was activated, while the other one was used as an internal control to compare the changes in dye intensity over time. The fluorescence in the spots was quantified before and after activation by summing the intensity of pixels in the ROI around each of the spots. Four mice were used in total in this work. All animal experiments were approved by Dartmouth IACUC, as outlined in the corresponding protocol # 00002170(a).

## 4. Conclusions

Overall, this study leveraged phase-changing perfluoropentane nanodroplets to act as laser-switchable NIR fluorescent probes. Loading a sufficient amount of DiR, a lipophilic dye, causes the nanodroplets to encapsulate dye in a quenched state, which decreases their fluorescence. However, after optically vaporizing the nanodroplets with NIR pulsed laser stimuli, microbubbles are formed, with a size up to 5× larger, which gives the DiR molecules more space and has them partially unquenched. This leads to an increase in the fluorescence intensity, which we captured here by NIR fluorescence imaging. Within this study, we tested the validity of using this platform to increase the ON/OFF signal upon activation by analyzing a range of DiR concentrations and establishing its functionality in a subcutaneous injection model.

## Data Availability

The data used and/or analyzed within this study are available from the corresponding author upon reasonable request.

## Supplementary Materials

The following supporting information can be downloaded at: https://www.mdpi.com/article/10.3390/ijms23137312/s1.
