# Supplementary material for: Fluorescent Phase-Changing Perfluorocarbon Nanodroplets as Activatable Near-Infrared Probes
Source: Int J Mol Sci. 2022 Jun 30;23(13):7312. doi: 10.3390/ijms23137312 (PMC9266996; doi:10.3390/ijms23137312)
Supplement: Supplementary file 1 [file ijms-23-07312-s001.zip › ijms-1758326-supplementary.pdf]

Fluorescent Phase-changing Perfluorocarbon Nanodroplets as Activatable Near-infrared Probes

Catalina-Paula Spatarelu, Austin Van Namen, Sidhartha Jandhyala and Geoffrey Luke

Supplementary Materials

DiR-loaded nanodroplets stability

In order to gain more insight into the stability of droplets, dynamic light scattering (Zetasizer, Malvern) was used. The size of nanodroplets in fetal bovine serum (FBS) and phosphate buffer solution (PBS) at 37 °C for a duration of 4h was investigated by taking a sample out for measurement every hour.

The size of nanodroplets did not vary too much in the first hour of the measurement (Fig. S1 A). The slight decrease of the nanodroplets could be due to phenomena such as Ostwald ripening, when some of the droplets will exchange the core, creating a population of droplets with lower size, and another population with larger sizes, that can then spontaneously vaporize due to lowering of the activation threshold with increasing size. A representation of this can be seen in the intensity-weighted size distribution of nanodroplets dispersed in FBS after 4h incubation (Fig. S1 E).

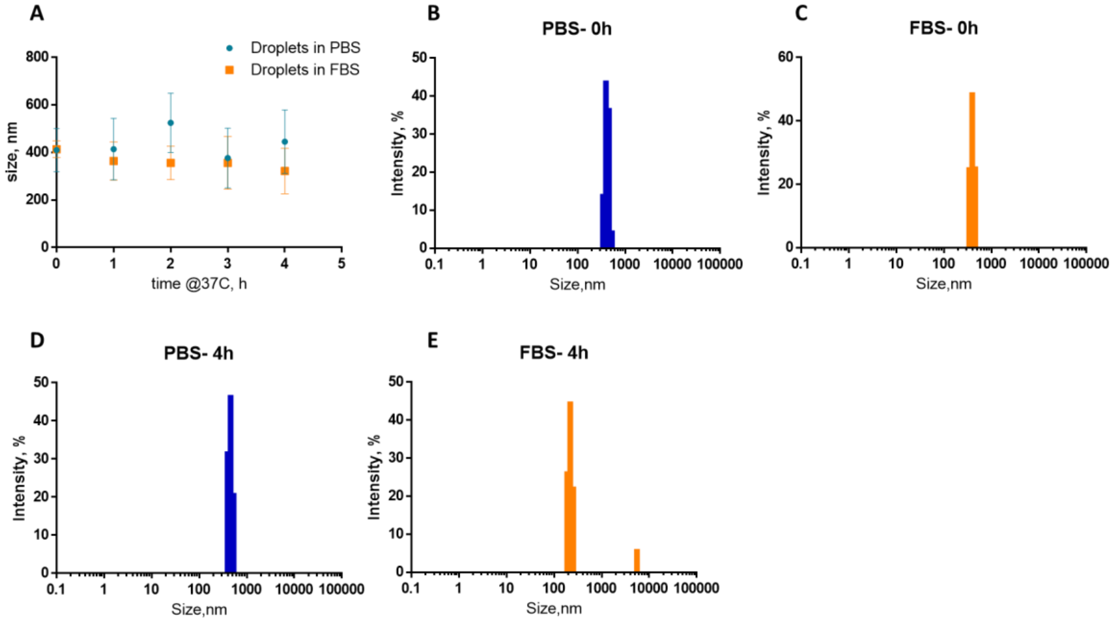

Figure S1. A. Average size of DiR-loaded nanodroplets incubated at 37 °C dispersed in PBS or FBS, respectively; B. Intensity-weighted size distribution of nanodroplets in PBS after synthesis; C. Intensity-weighted size distribution of nanodroplets in FBS after synthesis; D. Intensity-weighted size distribution of nanodroplets in PBS after 4h incubation at 37 °C; E. Intensity-weighted size distribution of nanodroplets in FBS after 4h incubation at 37 °C.

Quantum yield of DiR in various media

Fluorescent Phase-changing Perfluorocarbon Nanodroplets as Activatable Near-infrared Probes

Catalina-Paula Spatarelu, Austin Van Namen, Sidhartha Jandhyala and Geoffrey Luke

The ratio between the quantum yield of DiR in ethanol and in the presence of lipids was investigated using a method described before[1].

Briefly, a series of DiR concentrations was created by serial dilutions in ethanol and measured by means of UV-VIS (Varian Cary 50, Agilent) to determine the absorbance at 748 nm. The same samples were measured with the spectrofluorometer, using 748 nm excitation, and recording the emission between 765 nm and 900 nm. DiR-loaded liposomes were synthesized using the same composition as the nanodroplets, and sized through a syringe filter to achieve sizes smaller than 100 nm, and avoid scattering to influence the UV-VIS measurements. Following the same procedure as the ethanol samples, obtaining dilutions of liposomes in water and measuring both their absorbance as well as fluorescence. All settings were identical for the samples.

The computations of the quantum yield ratio between the two media was computed according to the following equation:

$$\frac{\varphi_{lipid}}{\varphi_{ethanol}} = \left(\frac{Gradient_{lipid}}{Gradient_{ethanol}}\right)\left(\frac{\eta_{lipid}^{2}}{\eta_{ethanol}^{2}}\right)$$

,where φ_{lipid} is the quantum yield of lipid-encapsulated DiR, and $\varphi_{ethanol}$ is the quantum yield of DiR in ethanol. The gradients are computed by linear regression of integrated fluorescence vs. absorbance data for the two media (Fig. S2), and the refractive indexes were obtained from literature, to be $\eta_{lipid} = 1.45$ and $\eta_{ethanol} = 1.3614$. [2-3]

Using this data, we obtained a ratio between the two quantum yields of 0.168.

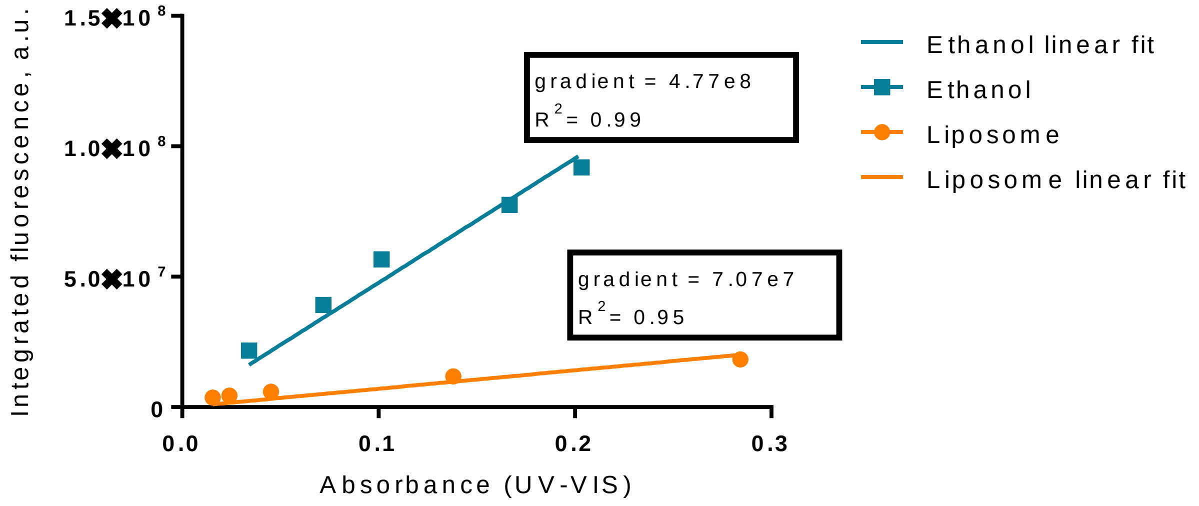

Figure S2. Integrated fluorescence against absorbance of samples of DiR in ethanol or liposome media, respectively.

Fluorescent Phase-changing Perfluorocarbon Nanodroplets as Activatable Near-infrared Probes

Catalina-Paula Spatarelu, Austin Van Namen, Sidhartha Jandhyala and Geoffrey Luke

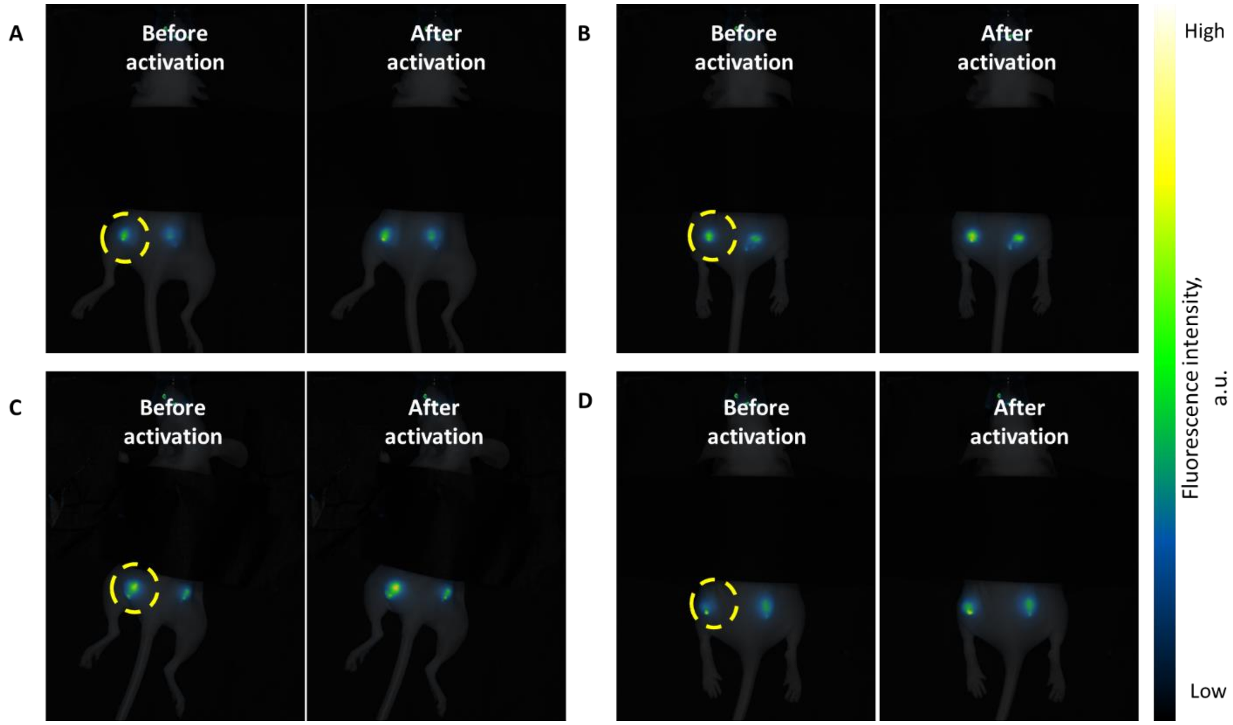

Figure S3. A-D. White images with fluorescence overlay of all mice injected with DiR-loaded nanodroplets in two spots in the hind legs, before and after activation of the left spot, respectively. All fluorescence images are displayed on the same colormap.

References

(1) HORIBA UK Limited A Guide to Recording Fluorescence Quantum Yields.

(2) Ardhammar, M.; Lincoln, P.; Nordén, B. Invisible Liposomes: Refractive Index Matching with Sucrose Enables Flow Dichroism Assessment of Peptide Orientation in Lipid Vesicle Membrane. *Proc Natl Acad Sci U S A* **2002**, *99* (24), 15313–15317. https://doi.org/10.1073/PNAS.192583499/ASSET/7DE1B511-188B-424E-AE4B-F129A5D27DD9/ASSETS/GRAPHIC/PQ2325834005.JPEG.

(3) *Refractive index of ethanol solutions.* http://www.refractometer.pl/refraction-datasheet-ethanol (accessed 2022-06-26).
